# Antibacterial Activity of Quantum-Confined One-Dimensional Titanate Nanofilaments

**DOI:** 10.1021/acs.langmuir.5c06846

**Published:** 2026-04-27

**Authors:** Mohammad Mozafari, Mohamed A. Ibrahim, Aidan McMoil, Jinjie He, Christopher M. Sales, Michel W. Barsoum, Masoud Soroush

**Affiliations:** † Department of Chemical and Biological Engineering, 6527 Drexel University, Philadelphia, Pennsylvania 19104, United States; ‡ Department of Materials Science and Engineering, 6527 Drexel University, Philadelphia, Pennsylvania 19104, United States; § Department of Civil, Architectural, and Environmental Engineering, 6527 Drexel University, Philadelphia, Pennsylvania 19104, United States; ∥ C&J Nyheim Plasma Institute, Drexel University, Camden, New Jersey 08103, United States

## Abstract

The emergence of antimicrobial resistance demands fundamentally new classes of antibacterial materials that operate through mechanisms distinct from conventional chemical or photodynamic pathways. Here, we introduce quantum-confined, one-dimensional lepidocrocite titanate nanofilaments (1DL NFs) as a previously unexplored inorganic nanomaterial platform that inactivates bacteria through direct contact-mediated membrane disruption. The 1DL-Ti NFs exhibit potent antibacterial activity against *Escherichia coli*, *Bacillus subtilis*, and *Listeria innocua*, achieving ∼96–99% inactivation within 4 h under ambient light and ∼85% in the dark, revealing light-independent efficacy. Multiparametric analyses—including reactive oxygen species assays, flow cytometry, and high-resolution electron microscopy—demonstrate a unique physical mechanism by which 1DL NFs result in membrane impalement, cell entrapment, and rapid biofilm-like agglomeration, distinct from ion- or reactive oxygen species-driven bactericidal pathways. Metal-ion release studies confirmed negligible leaching, ruling out ion-mediated toxicity. This “all-surface” architecture, enabled by the atomically thin one-dimensional structure of the NFs, differentiates them from conventional TiO_2_ nanocrystals and promotes strong interfacial contact with bacterial membranes. The synthesis is solution-based, low-temperature, highly scalable, and tolerant to the presence of several interlayer cations, providing modularity and manufacturability. These findings establish 1DL NFs as a new class of inorganic antibacterial materials with transformative potential for smart antimicrobial coatings, biomedical interfaces, water purification, and food-safety applications.

## Graphical abstract

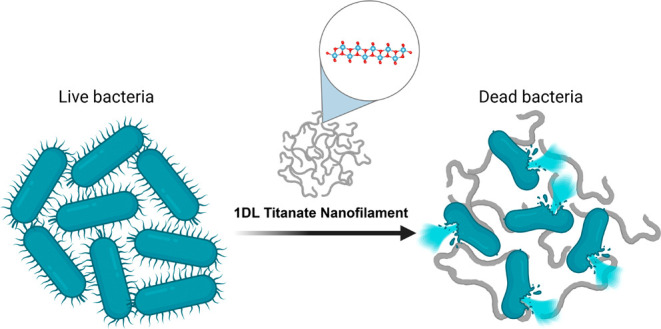

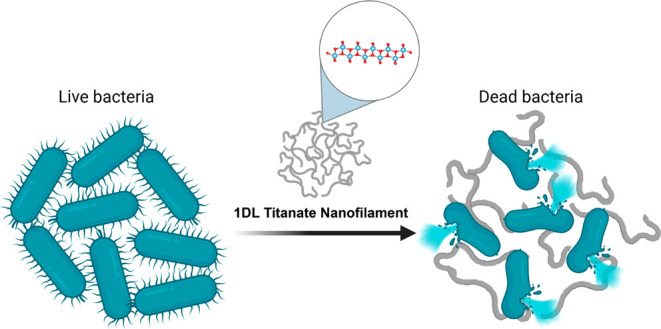

## Introduction

The escalating prevalence of antibiotic-resistant bacteria poses significant challenges to global healthcare and environmental safety. The rapid emergence of multidrug-resistant strains undermines the effectiveness of conventional antibiotics, necessitating innovative approaches to combat bacterial infections.^1^ Nanomaterials have emerged as promising candidates, offering broad-spectrum antibacterial (AB) activity through mechanisms such as membrane disruption, oxidative stress induction, and biofilm penetration.^2^ They can effectively target antibiotic-resistant strains and biofilm-associated infections, positioning them as potent agents for next-generation AB therapies.

Among advanced nanomaterials, titanium dioxide (TiO_2_) has gained significant attention as a versatile and eco-friendly option owing to its high chemical stability and safety. Several studies have proposed mechanisms behind its AB properties, including physical interactions between bacteria and nanomaterials, as well as the generation of reactive oxygen species (ROS). Upon light activation, TiO_2_ produces ROSs that effectively disrupt bacterial membranes, degrade biofilms, and inactivate pathogens.^3^ These characteristics, coupled with its excellent safety profile, render TiO_2_ a leading candidate for diverse applications, including healthcare disinfection and environmental remediation. Because of its remarkable properties, substantial efforts have been dedicated to synthesizing and tailoring TiO_2_ nanostructures to enhance their AB performance.^4^ However, conventional Ti-based AB materials, including anatase, rutile, P25 TiO_2_ nanoparticles, and TiO_2_ nanotubes, typically *require UV illumination* to reach meaningful AB efficiencies. Their activity under ambient light is greatly diminished, and their dark AB performance is generally minimal.^5^ Even TiO_2_ compositions modified with silver via sol–gel/hydrothermal synthesis, photodeposition, or chemical reduction still show limited intrinsic efficacy without light exposure.^6^ These limitations highlight the absence of a Ti-based antibacterial material that is both potent and light-independent, underscoring the need for fundamentally new Ti-based architectures capable of robust antibacterial performance under practical, low-light or dark conditions.

One-dimensional lepidocrocite titanate nanofilaments (1DL NFs) are a new class of nanomaterials that can be produced using a simple, one-pot, scalable method capable of producing the nanomaterials at the kilogram scale.^7–9^ This method involves reacting Ti-containing precursors with tetraalkylammonium hydroxides (TAAH) at atmospheric pressure and temperatures not exceeding 80 °C.^8,10,11^ These 1DL NFs consist of 2 × 2 TiO_6_ octahedra with cross sections of ∼ 5 × 7 Å^2^ and lengths of tens of nanometers (Figures S1a–1d),^10,12^ representing a lepidocrocite-type titanate structure distinct from conventional TiO_2_ polymorphs. The flocculation of the 1DLs upon the addition of cationic dyes to colloidal suspensions is a notable phenomenon.^13^ This immediate aggregation is visible to the naked eye and is primarily driven by electrostatic interactions, as the negatively charged 1DLs attract the positively charged dye molecules. In contrast, when anionic dyes are introduced, no flocculation occurs, underscoring the specificity of this interaction. This behavior has been observed in studies where 1DLs demonstrated high affinity for cationic dyes such as rhodamine 6G, crystal violet, and malachite green, with a maximum uptake exceeding 1,850 mmol.kg^–1^, 1,930 mmol.kg^–1^, and 2,061 mmol.kg^–1^, respectively, primarily via ion exchange mechanisms.

In this study, we present the first investigation into the AB activity of 1DL titanate NFs synthesized using tetramethylammonium hydroxide (TMAOH) or choline hydroxide (ChoOH). To assess the influence of intercalated cations on the AB properties, the original TMA^+^ cations are exchanged with potassium (K^+^) or sodium (Na^+^), creating K-1DL and Na-1DL variants. The AB activity of these materials is evaluated against model Gram-negative *Escherichia coli* (*E. coli*) and the Gram-positive *Bacillus subtilis* (*B. subtilis*) and *Listeria innocua* (*L. innocua*). We examine the antibacterial activity, ABA, in relation to NF size, incubation duration, and concentration, using colony-forming units (CFU) assays. To elucidate the potential AB modes-of-action, flow cytometry and reactive oxygen species (ROS) assays are further employed. Our findings suggest that 1DL NFs represent a new class of AB nanomaterials with significant potential applications in AB treatments and water purification.

## Experimental Section

### Materials Processing

The choice of the TAAH is pivotal for synthesizing and tuning 1DL NFs. These hydroxides dissolve titanium precursors into TiO_6_ octahedra and direct their growth into 1D structures, with the specific TAAH strongly influencing the final material. Smaller, hydrophilic TAAH such as TMAOH produce an interlayer spacing of 11.5 Å, whereas larger TAAH like TPAOH expand it to 14.8 Å, yielding less hydrophilic NFs that can suspend in organic solvents.^9^ Using ChoOH produces an environmentally benign 1DL material because choline is a naturally occurring essential nutrient,^14^ offering chemical stability and low toxicity rather than relying on toxic cations such as TMA^+^. Postsynthesis processing further shapes morphology: The self-assembly of 1DL NFs depends strongly on the solvent used during washing and drying.^15,16^ Washing TMA-1DLs with ethanol (EtOH) and drying yields porous mesostructured particles (PMPs) with spherical-like morphologies, whereas washing with water forms stable colloidal suspensions. Small-angle X-ray scattering shows that, in suspension, some 1DLs assemble into ribbons of ∼ 5–8 NFs, ∼ 300 Å long and one lepidocrocite sheet (≈5 Å) thick.^15^ These ribbons align along the *a*-axis, form nanobundles, and, upon drying, coalesce into sheets that stack along the *b*-axis. Stacking depends on interlayer cations: TMA^+^ induces ABABA ordering, while Li^+^ yields AAA stacking.^17^

A schematic illustration of the synthesis procedures for the 1DL NFs and Na- and K-PMPs is shown in Figure S2. TiB_2_ powder (as received, 99.9% purity, –325 mesh, Thermo Fisher Scientific Inc.) was mixed with either 25 wt % aqueous TMAOH (as received, 99.9999%, Thermo Fisher Scientific, USA) or 46 wt % aqueous ChoOH (as received, Thermo Fisher Scientific, USA) solutions in 250 mL polyethylene bottles. The TiB_2_-to-TMAOH/ChoOH molar ratio was kept at 0.6 because most of our results over the past 2 years were obtained at that ratio. The resulting material is thus well characterized and understood. Specifically, 10 g of TiB_2_ was added to 100 mL of TMAOH or 60 mL of ChoOH. The mixture was heated and agitated in an incubator (Labnet International Shaking Incubator, NJ, USA) at 180 rpm and 80 °C for 4 days with the bottles vented using two 23-gauge needles. (Caution: The reaction generates H_2_ and other gases that, if not vented, can explode violently) Postreaction, the resulting sediment was transferred to a 1 L beaker, mixed with EtOH (200 proof, Decon Lab), and stirred at ambient temperature for 5 min using an overhead mixer (OSC-10L-200 rpm, LabFish, China). The powder was allowed to settle, and the supernatant, containing excess TMA^+^/Cho^+^ cations and other EtOH-soluble byproducts, was discarded. This washing procedure was repeated several times until a neutral pH was achieved. This synthesis approach results in the formation of 1DL NFs through the reaction of TiB_2_ with TMAOH or ChoOH, as documented in recent studies.^9^

To prepare PMPs, the EtOH-washed sediments were allowed to dry in open air at 50 °C after the final EtOH wash. This drying process results in PMPs with spherical-like morphologies (see e.g. Figure 1c). Alternatively, to obtain colloidal suspensions, 15 mL of the EtOH-washed sediment was transferred into a 50 mL tube and centrifuged at 3500 rpm for 1 min to remove excess EtOH; the supernatant was discarded. Water was then added, and the material was resuspended by vortex shaking. After centrifugation at 5000 rpm for 1 h, a highly stable aqueous colloidal suspension was obtained, while any unreacted TiB_2_ settled at the bottom of the centrifuge tube and was discarded. The concentration of the colloidal 1DLs was determined by vacuum filtering 1 mL of the suspension through a 25 μm thick microporous monolayer polypropylene membrane (Celgard 3501, Celgard, NC, USA) using a fritted glass filter apparatus. The filtered films were fully dried in a vacuum oven at 80 °C overnight, and the weight of the residue was measured to quantify the 1DL colloidal concentration.

**Figure 1:**
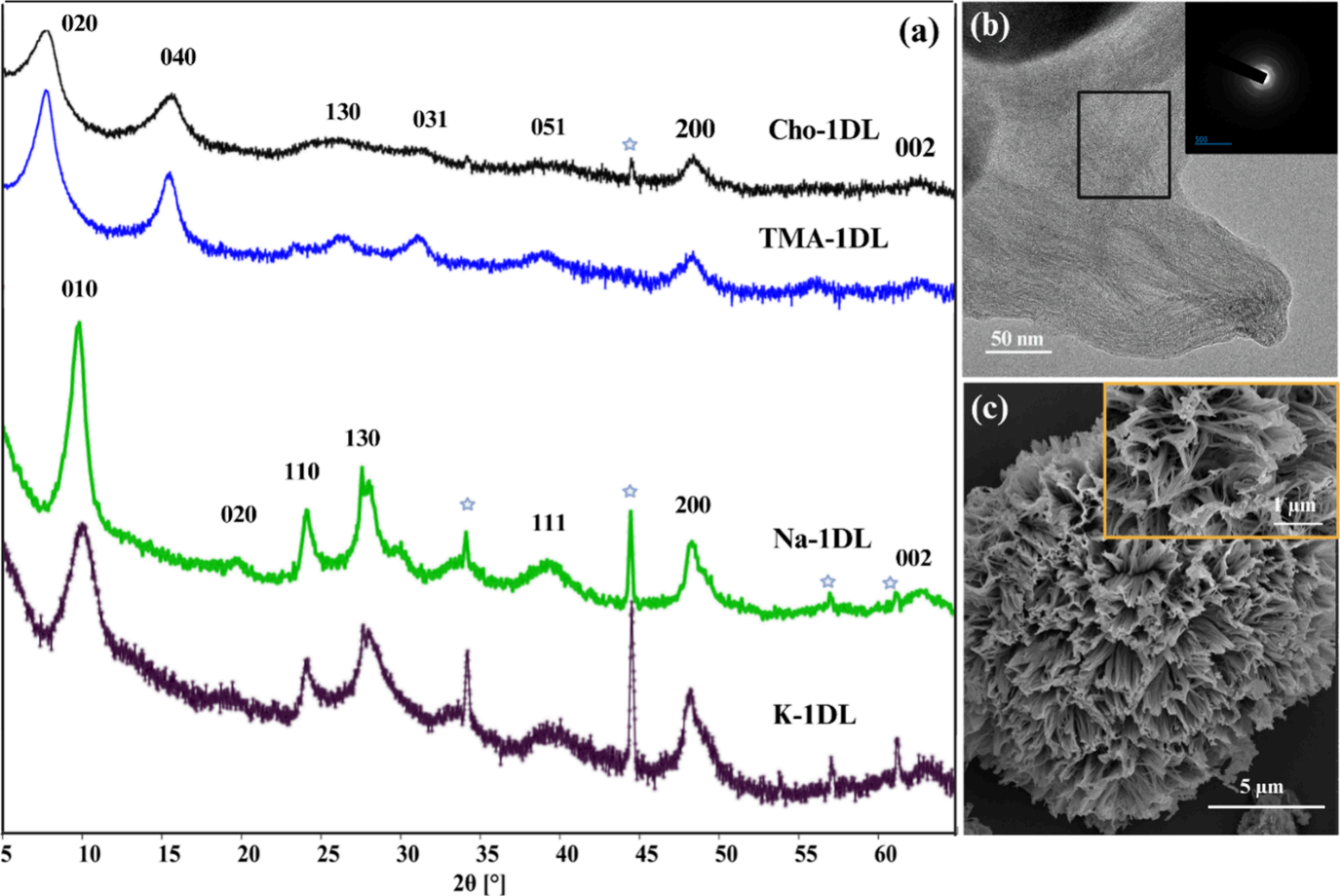
Characterization of 1DLs. (a) XRD patterns of Cho-1DL, TMA-1DL, Na-1DL, and K-1DL. Asterisks (*) indicate peaks corresponding to unreacted TiB_2_. (b) TEM image of the TMA-1DL colloidal suspension. (c) SEM image of Na-PMP. The Inset shows a larger magnification of the micrograph shown, where the 1D character is highlighted.

To synthesize K-1DL and Na-1DL PMPs, 2 g of EtOH-washed TMA-1DL sediment, while still wet, was placed in 50 mL of 0.5 M KCl or NaCl solution in a reaction bottle and stirred using a magnetic stirrer for 2 h. The mixture was transferred to a centrifuge tube for washing. The sediment was washed with DI water (for 4 cycles) to ensure the removal of excess salt. To enhance the dispersibility of K-1DL and Na-1DL, the washed, still-wet PMPs were transferred to a 100 mL glass beaker and sonicated using a probe sonicator (Fisherbrand Model 505 Sonic Dismembrator, Pittsburgh, USA) at 70% power, operating in intervals of 40 s on, and 10 s off, for a total duration of 40 min.

### Characterization

The TMA- and Cho-1DL colloidal suspensions were vacuum-filtered and dried at 50 °C overnight. The resulting filtered films, FFs, were finely ground into powders using a mortar and pestle, and X-ray diffraction (XRD) patterns were obtained from these crushed FF powders. For K^+^- and Na^+^-intercalated PMPs, XRD patterns were obtained from intercalated PMP powders after drying at 50 °C for 24 h. In all cases, the patterns were obtained using a diffractometer (Rigaku MiniFlex, Japan) equipped with a Cu K_α_ radiation source, operating at 40 kV and 15 mA. Scans were conducted over a 2θ range of 5°–65° with a step size of 0.02° and a dwell time of 1 s.

A scanning electron microscope (SEM) was used to observe the morphology and surface characteristics of the samples. Micrographs were acquired using a field emission scanning electron microscope (FE-SEM), (Zeiss Supra 50 VP, Carl Zeiss SMT AG Oberkochen, Germany). Prior to imaging, samples were sputter-coated with a platinum/palladium (Pt/Pd) layer for 30 s at 40 mA using a sputter coater (Cressington 208HR) to enhance conductivity and image quality. The SEM was operated at an accelerating voltage of 10 kV, utilizing the ’Inlens’ detector setting to optimize image clarity and detail.

A transmission electron microscope (TEM) was also used to image some of the samples. Images were acquired using a field-emission TEM (JEOL JEM2100F, Akishima, Tokyo, Japan), operated at an accelerating voltage of 200 keV, achieving an image resolution of approximately 0.2 nm. Images and diffraction patterns were recorded using a Gatan USC1000 CCD camera. To prepare samples for TEM analysis, approximately 10 μL aliquots of *E. coli* suspensions, with and without exposure to 1DL NFs, were drop-cast onto carbon-coated mesh grids. The grids were subsequently air-dried at room temperature for 2 h before TEM imaging.

### Bacteria Cell Preparation

*E. coli* (MG1655, Drexel College of Medicine, Gram-negative), *B. subtilis* (ATCC 6051, Gram-positive), and *L. innocua* (ATCC 51742, Gram-positive) were cultured in a Luria–Bertani (LB) medium at 37 °C overnight. The cultures were subsequently subcultured and harvested during the exponential growth phase. Cells were centrifuged at 5000 rpm for 10 min, and the resulting pellets were washed three times with phosphate-buffered saline (PBS, pH 7.4, Sigma-Aldrich, Germany) to remove residual medium and macromolecules. Finally, the pellets were resuspended in deionized (DI) water and diluted to achieve an approximate bacterial concentration of 10^8^ CFU/mL.

### Antibacterial Activity of 1DL

The AB activity against each strain was assessed using the CFU technique. The assays compared the AB efficacy of TMA-1DL, Cho-1DL colloids and Na-1DL, K-1DL PMPs. Bacteria, at a final concentration of 10^6^ CFU/mL, were incubated at 150 rpm and room temperature, with varying concentrations (200–2000 μg/mL) of TMA-1DL for different durations (0–5 h). After optimizing the concentrations and incubation times, the AB activity of the Na-1DL, K-1DL, and Cho-1DL samples was further evaluated. Subsequently, 100 μL of the cultures were plated on LB agar plates (Figure S3) and incubated for ∼ 18 h. The number of colonies formed was counted and compared with that of the control to calculate the bacterial inactivation percentage defined by

$$\mathrm{Inactivation}\;\mathrm{percentage}\;\left(\%\right)=\left[1-\left(\frac{A_{m}}{A_{c}}\right)\right]\times 100$$

where *A*_*m*_ and *A*_*c*_ represent the number of colonies on the 1DL-treated and control plates, respectively. Because the as-prepared 1DL colloidal suspensions are alkaline (pH ≈ 10), it was important to verify that the observed ABA was not a pH-driven artifact. Therefore, for all CFU assays, the 1DL colloids were diluted into PBS (pH 7.4) under identical conditions. The pH of the resulting bacteria–1DL mixtures was measured immediately after mixing (pH 7.47) and again after 4 h of incubation (pH 7.56). In both cases, the pH remained within the physiological range and was indistinguishable from that of the control samples containing only bacteria in PBS. All experiments were repeated three times, with three measurements taken per experiment; the average values are reported here.

### Flow Cytometry of Bacteria

Cells *of E. coli* and *L. innocua* at a concentration of 10^8^ CFU/mL were exposed to TMA-1DL at a final concentration of 1000 μg/mL, in a 96-well microtiter flat-bottom plate. The plate, containing both treated and untreated samples, was incubated at 150 rpm and RT for 4 h in complete darkness. Cell viability was assessed using flow cytometry (FC), (BD FAC Symphony A1, cell analyzer). For this, samples were incubated with 30 μL of propidium iodide for 15 min in the dark at RT. FC analyses were performed with a medium fluid rate, and a limit of 100,000 events was set for each trial. To minimize interference from debris, two thresholds were applied; forward angle scattering height (FSC-H) for signals greater than 10,000 and side-angle scattering height (SSC-H) for signals exceeding 100 were selected. Propidium iodide was excited with a 15 mW argon, Ar, ion laser (488 nm), and the fluorescence was detected using the FL2 channel, with a detection wavelength of 585 ± 40 nm. Fluorescence signals were amplified using the logarithmic mode and are displayed on a logarithmic scale. For single-cell analyses, an FSC-H vs FSC-A (A stands for the area of the signals) scatter plot was utilized to exclude doublets, which appeared as a distinct population with higher area values.

### Superoxide Radical (O^2•–^) Assay

The potential formation of superoxide radical anions (O^2•–^) by 1DL NFs was evaluated using the XTT reduction method. The tetrazolium salt XTT (2,3-bis­(2-methoxy-4-nitro-5-sulfophenyl)-2H-tetrazolium-5-carboxanilide, Fluka) is selectively reduced by O^2•–^ to form a water-soluble orange formazan product that exhibits a maximum absorption at 470 nm. A 0.4 mM XTT solution was prepared in phosphate-buffered saline (PBS, pH 7.0). Bacterial dispersions (1 mL) treated with 1DL NFs at different concentrations were mixed with 1 mL of the XTT solution and incubated for 0–4 h at RT in the dark. Following incubation, the suspensions were filtered through a 0.45 μm poly­(ether sulfone) (PES) membrane filter (Whatman) to remove residual NFs or other small solid particles. The absorbance of the filtrate was recorded at 470 nm using a UV–Vis spectrophotometer (Cary 60, Agilent Technologies, Santa Clara, CA, USA) at a scan rate of 300 nm·min^–1^. The increase in absorbance relative to the control (XTT solution without 1DL NFs) was used to estimate the relative O^2•–^ generation under the tested conditions. We also tested P25 (Evonik Industries) as a positive control.

### Quantification of Titanium Ion Release from 1DL NFs Using ICP-QQQ Analysis

To quantify potential Ti ion release from the 1DL NFs, an inductively coupled plasma triple quadrupole (ICP-QQQ) (8900 ICP-QQQ, Agilent Technologies, Santa Clara, CA, USA) mass spectrometer was utilized. Following incubation of aqueous suspensions of 1DL NFs with bacterial strains under conditions identical to those previously described, aliquots (0.5 mL) were collected and passed through a 0.22 μm syringe filter to remove bacterial cells and any aggregates. The filtered aliquots were then diluted to a final volume of 50 mL with ultrapure water, yielding a Ti-concentration of approximately 10 ppm, based on an assumed initial colloidal concentration of 1 mg/mL.

Calibration standards (10 ppm, 1 ppm, and 100 ppb) were prepared from a commercially available single-element Ti standard solution (Agilent 5190–8545, initial concentration of 1 mg/mL) using serial dilution. ICP-QQQ analyses were conducted using an ICP instrument (equipped with an SPS 4 autosampler, operating in helium (He) collision mode to minimize polyatomic interferences and optimize detection accuracy. DI water served as the analytical blank.

## Results and Discussions

### Characterization

Figure 1a displays typical XRD patterns of the materials investigated in this study. The top two patterns correspond to the Cho-1DL and TMA-1DL powders and exhibit notable similarities. In agreement with our previous work,^9^ the *d-spacings* calculated from the (020) peaks remained constant at ≈ 11.5 Å, attributed to the comparable sizes of TMA^+^ and Cho^+^ cations situated between the NFs. The patterns for the powders intercalated with Na^+^ and K^+^ are shown in the bottom two patterns in Figure 1a. Because of their smaller sizes, the *d*-spacings shrink to 9 Å. Note the presence of (130) peaks in the bottom two patterns indicates ABAB stacking.^17^ The *a*, *b*, and *c* lattice parameters are derived from the (200), (020), and (002) peaks, respectively (Figure 1a). Consistent with our previous findings, the *b* parameter is influenced by the nature of the intercalated cations, while the *a* and *c* parameters remain unaffected.^16^ The lattice parameters measured here, *a* = 3.76 Å and *c* = 2.97 Å, align with our prior work^10^ and are corroborated by the existing literature on lepidocrocite titanates.^18^

Sample SEM images of Na-PMP and K-PMP powders (Figures 1c and S4, respectively) show that both nanomaterials have the same semispherical, PMP morphology, composed of bundles of NFs exceeding 10 μm in lateral dimensions. The TEM image in Figure 1b confirms our previous findings^16,19^ that the colloid consists of quite fine NFs. The selected area electron diffraction, SAED, pattern (inset in Figure 1b) displays distinct arcs rather than complete rings, clearly underscoring the anisotropic, 1D nature of our material.

### Antibacterial Activity

Previous studies have reported that the size of a nanomaterial can have important ramifications for their ABA.^20^ To investigate this aspect, we bath-sonicated colloidal suspensions to produce 1DL NFs of varying sizes, hypothesizing that longer sonication times would yield smaller/shorter NFs while preserving their chemical composition and surface properties.^21^ Accordingly, TMA-1DL colloids were bath sonicated for 1.5, 3, and 8 h. The AB activity of each suspension was assessed against *E. coli* (final concentration of 10^6^ CFU/mL) using the CFU method, with a fixed TMA-1DL NF concentration of 1000 μg/mL incubated with *E. coli* for 3 h. As shown in Figure S5a, the percentage of *E. coli* inactivated increased from 71% for as-prepared TMA-1DL NFs to 92% for nanomaterials sonicated for 1.5 h. Further sonication did not increase this value and, unless otherwise noted, this is the sonication time used throughout. The lack of further enhancement with longer sonication times remains unclear and warrants additional investigation. The 1DL NFs intercalated with choline, K^+^, and Na^+^ were also subjected to 1.5 h of sonication under the same conditions. This trend may be attributed to changes in NF morphology. The sharp edges formed during sonication likely play a critical role in disrupting and penetrating bacterial cell walls, leading to cell damage and enhanced ABA.^20^ Similar observations have been reported with nanomaterials like graphene oxide and carbon nanotubes, where sharp edges or tubular structures insert into lipid bilayers, creating pores and disrupting membrane continuity, ultimately causing bacterial lysis.

To optimize the incubation times, 1.5 h-sonicated TMA-1DL (at 1000 μg/mL) was tested against *E. coli* (10^6^ CFU/mL) over 0 to 5 h. As illustrated in Figure S5b, bacterial inactivation efficiency increased steadily with longer incubation times, reaching approximately 96% after 4 h. Beyond this point, no significant improvement was observed, and at later time point, inactivation efficiency decreased. This suggests that prolonged contact between NFs and bacterial cells enhances the AB effect, likely due to increased interaction or accumulation of reactive species at the bacterial surface. However, the decline observed beyond 4 h could be attributed to bacterial response mechanisms, such as the production of protective metabolites or activation of biological pathways that mitigate the AB effect. Additionally, an equilibrium may be established where the rate of bacterial killing is offset by bacterial growth, diminishing overall efficacy.^22^

We further assessed the AB activity of TMA-1DL NFs against *E. coli*, *B. subtilis*, and *L. innocua* at concentrations of 200, 500, 800, 1000, and 2000 μg/mL, following 4 h of incubation using the CFU method. Agar plate photographs (Figure 2) clearly demonstrate a reduction in bacterial colonies with increasing TMA-1DL concentrations. Quantitative analysis (Figure 3a) revealed that *E. coli* exhibited a pronounced concentration-dependent inactivation, increasing significantly from 20.5% at 200 μg/mL to a maximum of 96.2% at 1000 μg/mL, with a slight decrease at higher concentrations. In contrast, *B. subtilis* showed moderate concentration dependence, with inactivation efficiency increasing from approximately 89% at 200 μg/mL to about 99% at 1000 μg/mL. *L. innocua* displayed minimal concentration dependence, maintaining consistently high inactivation efficiencies (99%) across all concentrations. These observed differences between bacterial strains may be attributed to variations in their cell wall structures and/or compositions. *E. coli*, a Gram-negative bacterium, possesses an outer membrane that can act as a barrier to AB agents, potentially requiring higher concentrations of NFs for effective inactivation. Conversely, *B. subtilis* and *L. innocua*, both Gram-positive bacteria, lack this outer membrane, which may render them more susceptible to NF-induced membrane disruption, even at lower concentrations. Similar patterns of differential susceptibility have been reported in studies investigating the AB effects of natural compounds. For instance, vanillin exhibited varying minimum inhibitory concentrations (MICs) against *E. coli* and *L. innocua*, highlighting species-specific responses to antimicrobial agents. These findings underscore the importance of considering bacterial cell wall characteristics when evaluating the efficacy of nanomaterial-based AB agents.

**Figure 2:**
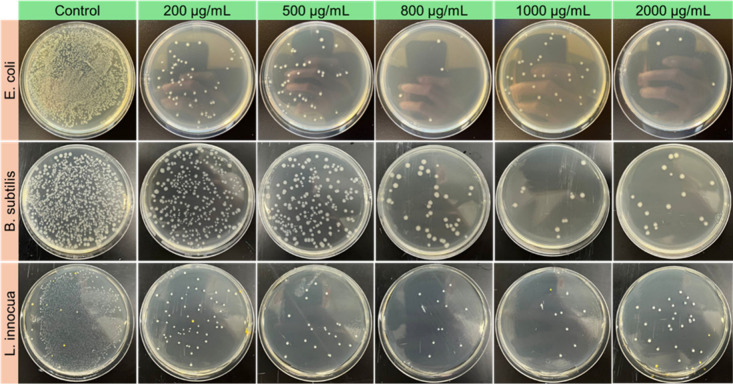
Concentration-dependent AB activity of TMA-1DL NFs. Photographs of agar plates showing *E. coli*, *B. subtilis*, and *L. innocua* bacterial cells (final concentration 10^6^ CFU/mL) recultivated after 4 h of treatment starting at the concentrations of TMA-1DL indicated. Bacterial suspensions (final concentration 10^6^ CFU/mL) in DI water without TMA-1DL material served as controls.

**Figure 3:**
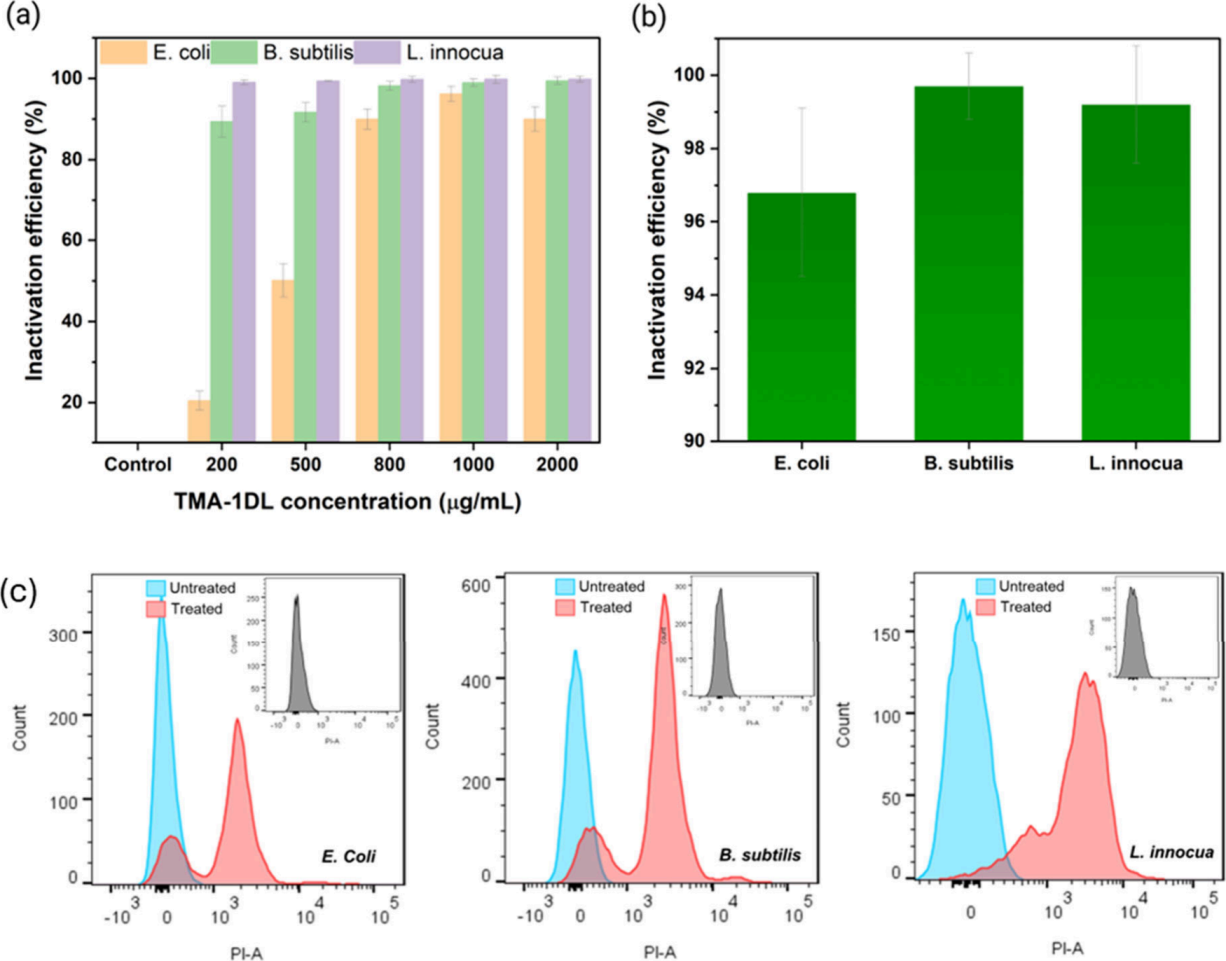
AB activity against *E. coli*, *B. subtilis*, and *L. innocua* (10^6^ CFU/mL) after 4 h of incubation. CFU assay showing the effect of (a) TMA-1DL NFs at various concentrations and (b) Cho-1DL at 1000 μg/mL. (c) Flow cytometry analysis of bacteria exposed to 1000 μg/mL TMA-1DL in the dark. Histograms depict bacteria treated with TMA-1DL (red) and untreated control (blue). Insets show autofluorescence histograms of unstained bacteria for comparison.

In summary, TMA-1DL NFs derived from pulverizing FFs exhibit excellent AB properties. Upon introducing TMA-1DL suspensions to bacterial cultures, visible agglomeration occurred almost immediately (Figure S6). This phenomenon is likely due to the presence of dissolved ions in PBS, such as Na^+^, Cl^–^, K^+^, and phosphate, which increase ionic strength and can induce flocculation. Notably, when deionized (DI) water was used instead of PBS, flocculation did *not* occur, suggesting that ionic strength indeed plays a crucial role in NF aggregation. AB assays conducted in DI water, as shown in Figure S7a–7b, demonstrated inactivation efficiencies of 91%, 93.7%, and 96.8% for *E. coli*, *B. subtilis*, and *L. innocua*, respectively. These values are slightly lower than those observed in PBS, potentially due to the absence of flocculation in DI water, which may reduce direct interactions between the bacteria and the NFs. The higher ionic strength in PBS could enhance NF aggregation, increasing contact with bacterial cells and leading to higher inactivation efficiencies. Furthermore, some cells within these aggregates might be in a viable but nonculturable state, affecting the observed AB activity. These findings underscore the importance of medium composition and ionic strengths in modulating the AB efficacy of nanomaterials.

This attraction of the bacteria to 1DLs is somewhat unexpected because, at pH 7.4, the zeta potentials of *E. coli* cells (∼ – 30 mV),^23^ *B. subtilis* (∼ – 15 mV),^24^ and *L. innocua* (∼ – 20 mV)^25^ have the same sign as the 1DL zeta potentials (−50 mV range^19^), with all values being negative. This similarity suggests that simple electrostatic repulsions should prevent electrostatic attractions between the bacteria and 1DL NFs. However, the observed agglomeration suggests that electrostatic screening by cations in PBS reduces repulsive interactions, allowing van der Waals forces, hydrophobic effects, or specific binding affinities to promote aggregate formation. These aggregates could be easily filtered out in PBS. Further research is necessary to elucidate the exact mechanisms underlying this phenomenon, but such investigations are beyond the scope of this paper.

In pursuit of developing environmentally sustainable AB agents, we synthesized 1DL NFs using ChoOH instead of TMAOH. As shown in Figure 3b, the resulting Cho-1DL NFs demonstrated significant AB efficacy, achieving inactivation efficiencies of approximately 96.8% for *E. coli* and 99% for both *B. subtilis* and *L. innocua*. Agar plate images (Figure S8a) clearly illustrate the reduction in bacterial colonies across all three strains. Additionally, visible agglomeration in the treated bacterial suspensions was observed (Figure S8b), again indicating some interactions between the NFs and bacterial cells. These findings are particularly significant as they suggest that environmentally benign 1DL NFs can be readily produced using ChoOH, offering a safer alternative to TMAOH-based synthesis methods. This aligns with previous studies highlighting the AB properties of choline-based compounds.^26^ For instance, choline carboxylic acid–based ionic liquids have demonstrated notable AB activity, attributed to their ability to disrupt microbial cell membranes.^27^ Moreover, cholinium-based ionic liquids have been shown to possess excellent AB properties, comparable to standard antibiotics like streptomycin.^28^ The successful synthesis of Cho-1DL NFs not only imparts environmentally benign characteristics to the materials but also broadens their potential applications in biomedical fields, particularly in applications where nontoxic but effective AB agents are required.

In our final set of experiments, we explored the potential of producing nontoxic PMPs by ion-exchanging TMA^+^ cations in 1DL NFs with Na^+^ and K^+^. The resulting Na-1DL and K-1DL PMPs demonstrated AB efficacy comparable to their TMA^+^-intercalated counterparts, as evidenced by the results presented in Figure S9. Notably, due to their relatively larger size, these PMPs did not remain suspended for the 4 h duration of the AB assays. However, despite sedimentation, they maintained high AB activity, suggesting that wet 1DL sediments in the form of PMPs could effectively inactivate bacteria. This observation opens the possibility of developing 1DL-based AB gels. Recent studies have shown that adding a few drops of acid to 1DL colloidal suspensions induces rapid gelation, resulting in hydronium-cross-linked inorganic hydrogels with good compressive strengths.^29^ We have also shown that simply adding common salt solutions to 1DL colloids can result in their gelation.^30^ The ability to ion-exchange TMA^+^ with environmentally benign cations without compromising AB efficacy, coupled with the potential to form robust hydrogels, underscores the versatility of our materials. These findings suggest promising applications in environmental fields, particularly in developing antibacterial coatings and water purification systems.

Flow cytometry (FC) analyses were conducted on *E. coli*, *B. subtilis*, and *L. innocua* following a 4 h treatment with 1000 μg/mL of TMA-1DL NFs in the dark. The FC results, presented in Figure 3c, reveal a substantial reduction in live bacterial cells post-treatment. Specifically, the percentage of live cells decreased from 97.2% to 15.1% for *E. coli*, from 98.1% to 12.9% for *B. subtilis*, and from 98.9% to 8.5% for *L. innocua*. These significant decreases indicate that our material can effectively damage bacterial cell membranes, leading to cell death even in the *absence* of light. It is important to note that FC analysis detects cells with compromised membranes as alive, whereas the CFU method considers them nonviable due to their inability to proliferate in growth media. This methodological difference accounts for the observed discrepancies in viability assessments between the two techniques.^31^ Nonetheless, both methods conclusively demonstrate that 1DL NFs possess potent AB properties in the dark, distinguishing them from traditional titanates and TiO_2_, that require light activation for similar efficacy.^32^

The slightly lower AB efficiency observed against *E. coli* compared to *B. subtilis* and *L. innocua* can again be attributed to differences in their cell wall structures.^33^ *E. coli*, a Gram-negative bacterium, possesses a thinner peptidoglycan layer (7–8 nm) covered by an external protective lipid membrane, which provides additional resistance to antimicrobial agents. In contrast, Gram-positive bacteria like *B. subtilis* and *L. innocua* have thicker peptidoglycan layers (20–80 nm) but lack the external lipid membrane,^34^ making their cell walls more susceptible to damage by direct contact with the 1DL nanostructured surfaces.

SEM images of the agglomerates (Figure 4) offer valuable insights into the AB mechanisms of 1DL NFs. The images depict bacteria physically entrapped or wrapped by the nanometer-thin 1DL NFs, leading to the formation of agglomerates. This suggests that at elevated concentrations, 1DL NFs likely entangle bacterial cells, restricting their mobility and resulting in their inactivation/death. Additionally, the sharp 1DL NF edges probably result in significant membrane damage, compromising cellular integrity and contributing to bacterial death.^35^ Further SEM analysis (Figure 5) of bacterial cells treated with 1000 μg/mL TMA-1DL NFs reveals extensive cell lysis, characterized by severe membrane disruption and cytoplasmic leakage (highlighted by red arrows). These morphological alterations indicate direct interactions between the NFs and bacterial cells, leading to detachment of the cytoplasmic membrane from the cell wall and subsequent cellular collapse. Such observations align with the quantitative reductions in bacterial viability observed in both CFU assays and FC analyses (Figures 2 and 3c, respectively).

**Figure 4:**
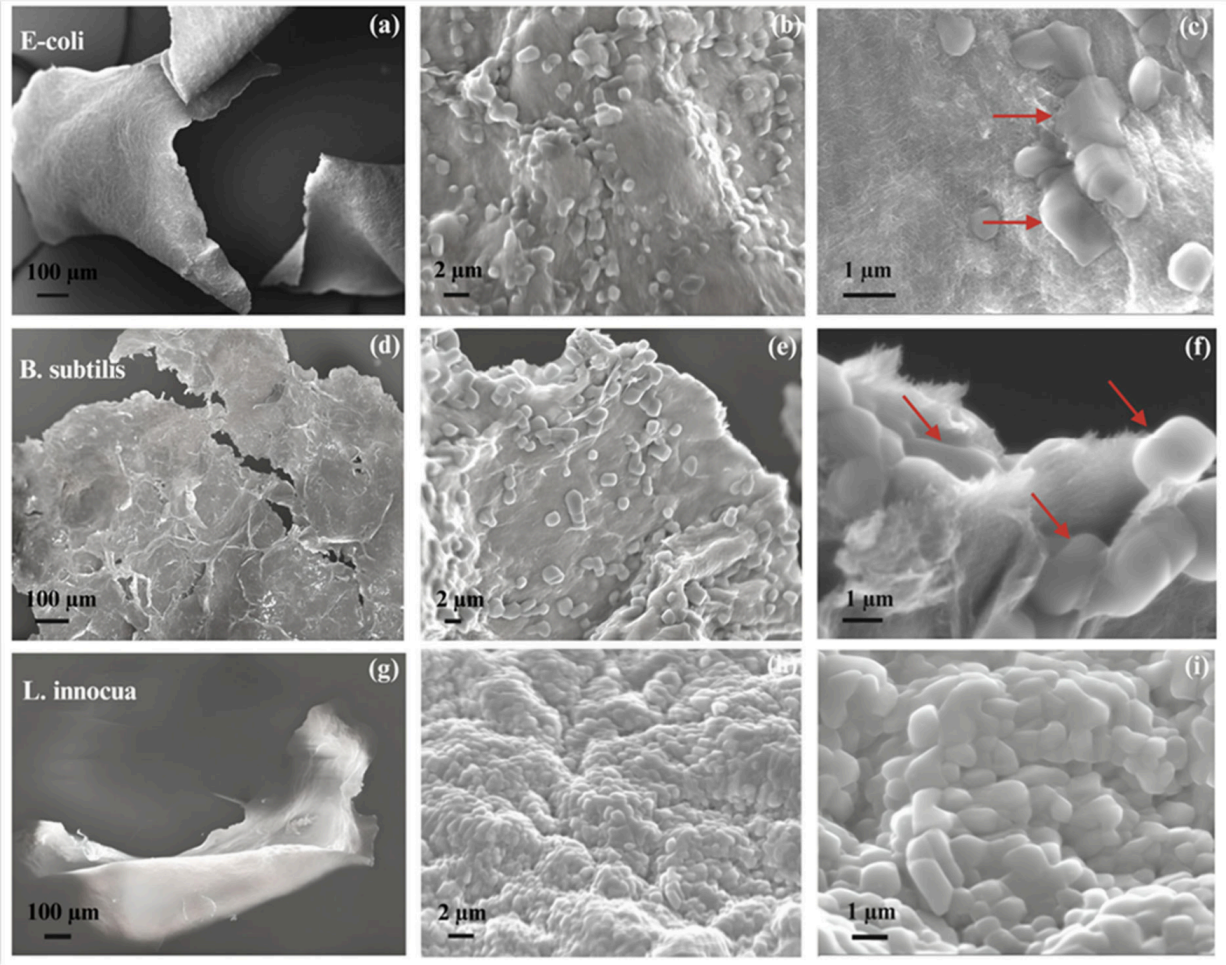
SEM images of bacterial aggregates formed through interaction with TMA-1DL colloid at different magnifications: (a–c) *E. coli*, (d–f) *B. subtilis*, and (g–i) *L. innocua* aggregates. High 1DL specific surface area enables efficient bacterial capture and aggregation.

**Figure 5:**
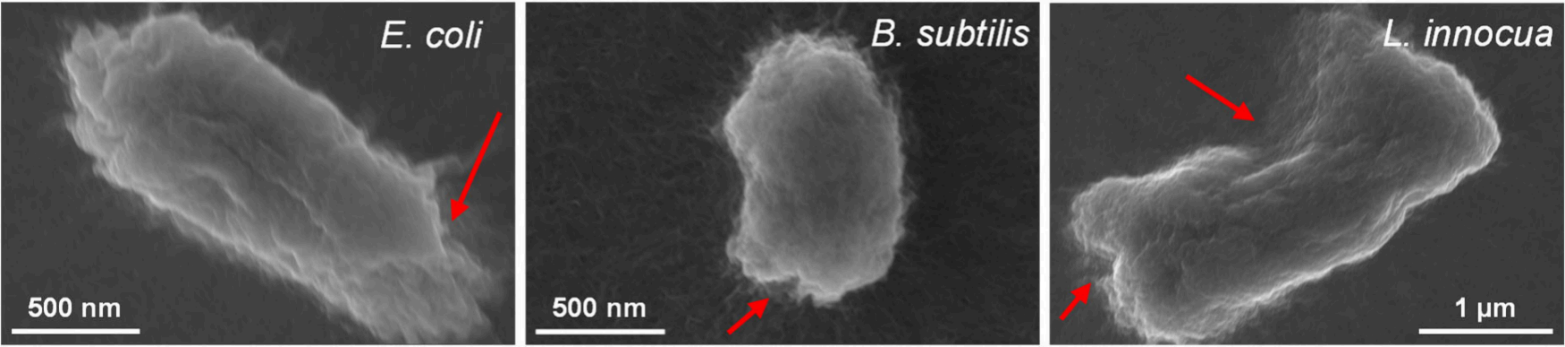
SEM images of bacteria treated with 1000 μg/mL of TMA-1DL NFs. The treated cells exhibit extensive cell lysis, characterized by severe membrane disruption and cytoplasmic leakage (indicated by red arrows).

To provide additional evidence for direct membrane disruption as the primary AB mechanism, we used a TEM to examine morphological changes in *E. coli* bacterial cells before and after exposure to the NFs. The untreated bacteria (Figure 6a) exhibited intact cell membranes and well-defined morphologies. In contrast, significant morphological damage was evident upon treatment with 1DL NFs (Figures 6b and 6c), characterized by disrupted cell membranes and visible entanglement with the NFs. These findings visually corroborate our SEM observations (Figure 5) and strongly support the conclusion that the ABA of the 1DL NFs predominantly arises from direct physical interactions with bacterial cell membranes.

**Figure 6:**
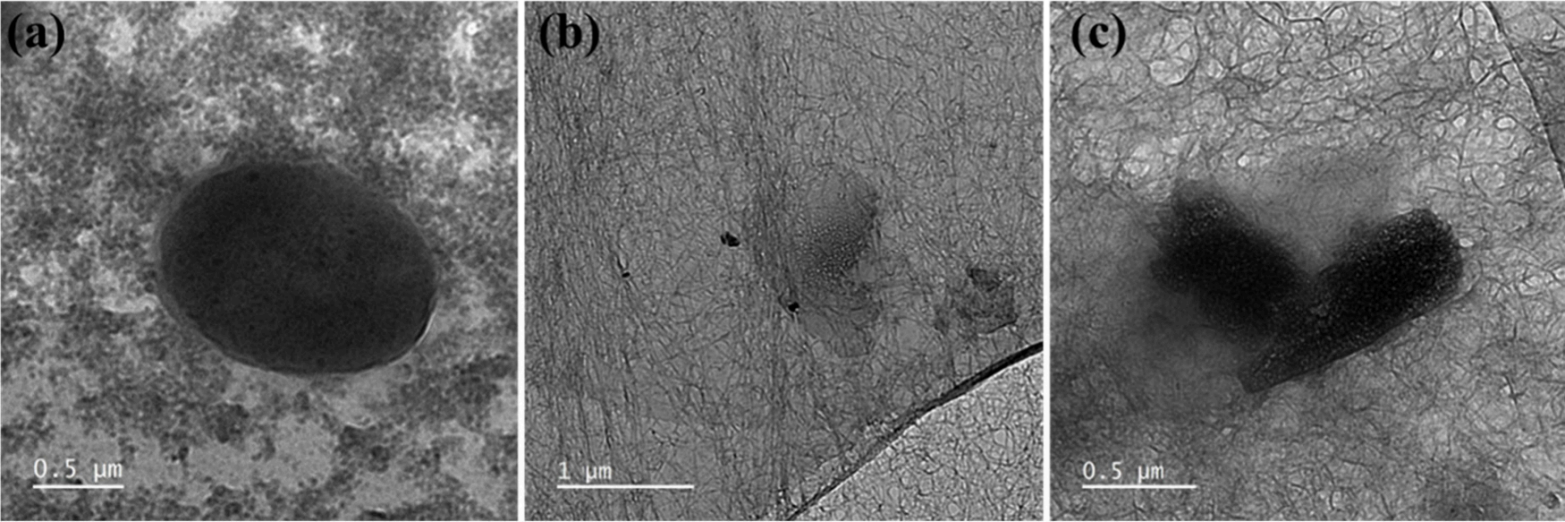
TEM images of *E. coli*: (a) untreated (control) showing intact bacterial cell membranes and a uniform morphology, (b) and (c) after treatment with 1DL NFs, showing significant membrane disruption presumably induced by direct physical interactions.

In past work where AB activity was observed in the dark, the presence of ROS was frequently invoked as the primary mechanism for bacterial destruction (Table S1). Because oxidative stress is widely proposed as a dominant AB mechanism for many nanomaterials containing metals, metal oxides, or carbon structures,^36^ it was reasonable to question whether a similar ROS-mediated mechanism could explain the AB activity of our 1DL NFs. To test this possibility, we quantified ROS generation in comparison with P25. Note that in the 1DL system, ROS production would be proportional to any Ti cations with a charge < + 4, e.g. Ti^3+^. However, we have previously shown that even if Ti^3+^ cations are present initially, they rapidly oxidize to +4.^37^ Said otherwise if ROS are generated in the dark, their effect would be transient at best. To determine whether oxidative stress contributes to the AB activity of 1DL NFs, the generation of O^2•–^ was evaluated using an abiotic XTT reduction assay.^35^ As shown in Figure S10, P25 (1000 μg/mL) exhibited a noticeable increase in absorbance at 470 nm over time, confirming its tendency to generate a small amount of O^2•–^. In contrast, all 1DL NF samples, even at concentrations up to 2000 μg/mL, showed *no* measurable change in absorbance, indicating no detectable O^2•–^ generation. Despite the absence of detectable ROS production, our results demonstrate that TMA-1DL NFs exhibit substantial ABA in the dark, achieving reductions of 84.9%, 87.1%, and 91.5% against *E. coli*, B. subtilis, and L. innocua, respectively (Figure S11). Given the environmentally benign nature of titanate-based materials, these findings strongly support direct membrane disruption, rather than ROS-mediated oxidative stress, as the dominant AB mechanism of 1DL NFs. This insight not only distinguishes 1DLs from conventional photoactivated titania-based AB agents but also expands their potential applications where light exposure is limited or undesirable, although the possible involvement of other ROS types cannot be fully excluded at this time.

Ion release from AB nanomaterials has also been recognized in the literature as a potential mechanism contributing to bacterial inactivation.^38^ To assess whether ionic dissolution contributes to the observed AB activity, inductively coupled plasma (ICP) analysis was conducted on aqueous suspensions of the 1DL NFs incubated under identical experimental conditions with each bacterial strain. The ICP results revealed minimal Ti ion release into the solution after 4 h of exposure, specifically 4.218, 0.406, and 0.21 ppb for suspensions containing *L. innocua*, *B. subtilis*, and *E. coli*, respectively. Given these very low Ti concentrations, it is reasonable to conclude that dissolved Ti is not a contributor to the AB activity. These findings further substantiate that direct physical membrane disruption is indeed the primary AB mechanism exerted by the 1DL NFs.

As summarized in Table S1, a range of Ti-based AB materials based on anatase, rutile, and P25 can achieve high bacterial inactivation under optimized, strongly illuminated conditions, with several reports approaching ≈94–99% reduction of bacterial strains at loadings on the order of 100–1000 μg/mL. However, their performance under ambient light is markedly more variable, and in the dark the reported inactivation efficiencies generally fall to the ≈60–70% range at comparable concentrations and cell densities. *By contrast, under comparable conditions, our 1DL NFs achieve 96–99% inactivation under ambient light and 85–92% in the dark, representing the highest intrinsic, light-independent AB efficacy reported for any Ti-based nanomaterial to date.* This stark performance gap, clearly reflected in Table S1, underscores that the antibacterial activity of 1DL NFs is primarily governed by contact-mediated physical membrane disruption rather than the ROS-dependent pathways characteristic of other TiO_2_-based systems, thereby enabling strong AB activity even in the absence of photoactivation.

The highly hydrophilic surfaces of 1DL NFs likely facilitate close interfacial contact with bacterial membranes, thereby enhancing direct physical interactions. This intimate contact presumably allows the nanometer-thin, sharp-edged NFs to exert mechanical stress on the cell envelope, leading to localized deformation, membrane rupture, and cytoplasmic leakage.^39^ These findings provide compelling evidence that the ABA of 1DL NFs primarily arises from direct physical disruption of bacterial membranes rather than from electrostatic or chemical effects. While the term “quantum-confined” describes the atomically thin, one-dimensional nature of these titanate nanofilaments, the present results do not identify quantum confinement itself as an independent AB mechanism. Since both bacterial surfaces and 1DL NFs are negatively charged under physiological conditions, long-range electrostatic attraction is unlikely. Instead, the nanoscale thickness, high aspect ratio, and atomically sharp edges of the 1DL NFs presumably enable efficient physical engagement with bacterial envelopes upon contact. This structural penetration mechanism explains the strong AB activity observed even under dark conditions and is consistent with the minimal Ti ion release detected by ICP analysis. Collectively, these observations confirm that mechanical membrane disruption, rather than charge perturbation or photoinduced ROS generation, is the dominant AB pathway for 1DL NFs.

It is important to note that the ABA of 1DL NFs primarily arises from direct physical disruption of bacterial membranes rather than from chemical consumption of the active material; consequently, the NFs are not inherently depleted during their AB action. As noted above, the resulting aggregates can be easily removed by filtration, and the ability of these materials to form hydronium-cross-linked inorganic hydrogels with appropriate compressive strengths^29^ or to undergo gelation upon the addition of common salt solutions to 1DL colloids^30^ provides straightforward pathways for material recovery after use. The practical feasibility of reuse, including potential fouling by bacterial debris and possible loss of NF structural integrity during repeated AB cycles, warrants further investigation.

Further research is needed to fully understand the interactions between the 1DL NFs and bacterial membranes and to evaluate the potential environmental and health impacts associated with residual NFs in aqueous systems. Such studies are crucial for the development of safe and effective antimicrobial technologies. Additionally, the simplicity and scalability of the 1DL NF synthesis are noteworthy. Our laboratory has successfully produced kilogram-scale quantities of free-flowing PMP powders under ambient pressure and at temperatures below 80 °C, with nothing more sophisticated than plastic bottles and hot plates. This straightforward and cost-effective production approach, based on abundant and earth-common elements, offers significant advantages over many existing materials for biomedical and industrial applications. Importantly, 1DL colloidal dispersions are practically relevant to applications such as water disinfection and microbial control in aqueous systems, and they also serve as precursor suspensions for AB gels, coatings, and composite materials.^40^

Beyond dye interactions, 1DL PMPs also exhibit remarkable capabilities in water purification, particularly in the adsorption of heavy metals. They rapidly adsorb actinides such as uranium (U^4+^) and thorium (Th^4+^), with adsorption capacities reaching up to 424 mg/g for U^4+^ and 292 mg/g for Th^4+^. These substantial capacities highlight the potential of 1DLs for transforming contaminated water into potable water.^41^

## Conclusions

In summary, this work establishes quantum-confined, 1DL titanate NFs as a new class of inorganic AB materials that achieve ∼96–99% inactivation of both Gram-negative and Gram-positive bacteria under ambient light and ∼85–92% in the dark. This performance surpasses that of previously reported Ti-based AB materials under comparable conditions. The exceptional, light-independent efficacy arises from a structurally encoded rather than chemically activated mode of action, eliminating the UV dependence that has long limited conventional TiO_2_-based systems. Notably, the interlayer cation composition can be varied among TMA^+^, K^+^, Na^+^, and choline without compromising AB performance, offering a level of chemical tunability uncommon for physical-mechanism nanomaterials. Combined with the demonstrated capacity for actinide capture, compatibility with hydrogel and coating formulations, and kilogram-scale accessibility from earth-abundant precursors, these NFs represent a versatile and scalable platform for next-generation water purification and antimicrobial surface technologies.

## Supplementary Material



## Notes

Structural model of the one-dimensional titanate nanofilaments; schematic of the synthesis procedures; description of the colony-forming unit counting method; additional SEM images; optical images and agar plate photographs of recultivated bacterial cells; results of superoxide radical formation assays; and comparison of the antibacterial activity of titanate nanofilaments with TiO_2_-based materials reported in the literature (PDF)
