# Supplementary material for: Antibacterial Activity of Quantum-Confined One-Dimensional Titanate Nanofilaments
Source: Langmuir. 2026 Apr 27;42(18):12509–19. doi: 10.1021/acs.langmuir.5c06846 (PMC13178067; doi:10.1021/acs.langmuir.5c06846)
Supplement: Supplementary file 1 [file la5c06846_si_001.pdf]

Supporting Information (SI)

Antibacterial Activity of Quantum-Confined One-dimensional Titanate Nanofilaments

Mohammad Mozafari[1,‖], Mohamed A. Ibrahim[2,‖], Aidan McMoil[2], Jinjie He[3], Christopher M. Sales[3], Michel W. Barsoum[2,*], and Masoud Soroush[1,2,*]

[1] Department of Chemical and Biological Engineering, Drexel University, Philadelphia, Pennsylvania 19104, USA

[2] Department of Materials Science and Engineering, Drexel University, Philadelphia, Pennsylvania 19104, USA

[3] Department of Civil, Architectural, and Environmental Engineering, Drexel University, Philadelphia, Pennsylvania 19104, USA

Submitted for Publication in *Langmuir*

March 31, 2026

Keywords: *antibacterial activity; one-dimensional nanomaterials; titanate nanofilaments; E. coli; B. subtilis; L. innocua; quantum confinement*

‖ Mohammad Mozafari and Mohamed A. Ibrahim contributed equally to this work.

* Corresponding authors: barsoumw@drexel.edu, and soroushm@drexel.edu

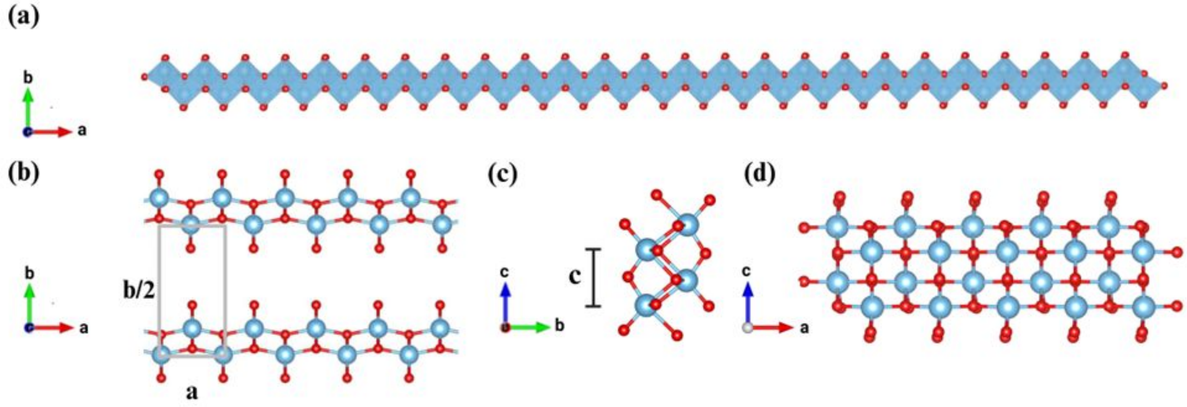

Figure S1. Characterization of 1DLs. (a) DFT model of 1DL structure showing 2 × 2 sharing edges TiO_6 octahedra growing along [100], (b) the a-b plane depicting a and b lattice parameters, (c) the b-c plane showing the c lattice parameter, and (d) the a-c plane[1].

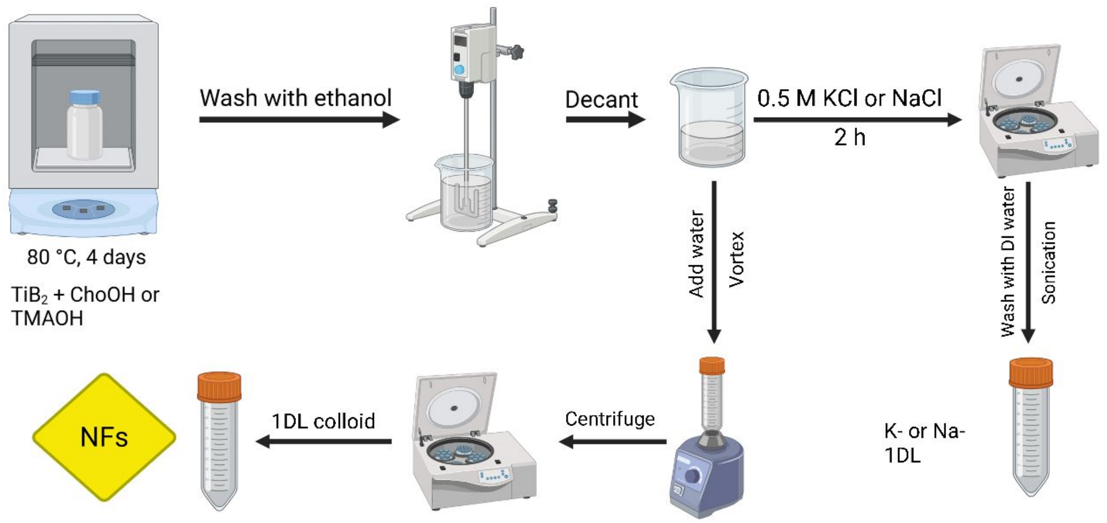

Figure S2. Schematic illustration of the synthesis procedures for 1DL NFs and Na- and K-PMPs.

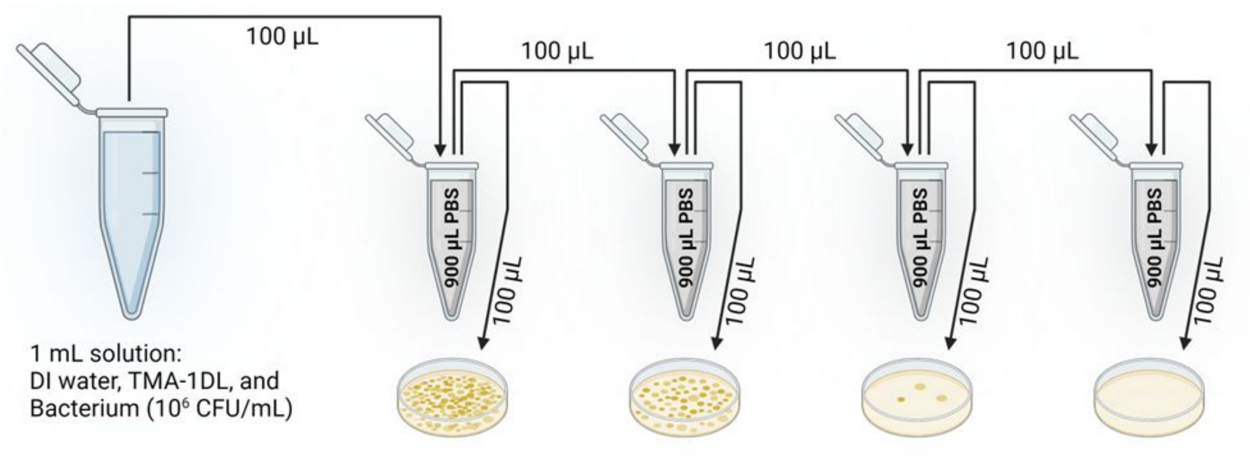

Figure S3. Illustration of colony-forming unit (CFU) counting process. Cells from untreated and 1DL NFs treated samples are serially diluted (1:4), and 100 µL of each dilution is spread on agar plates to form colonies overnight. The colonies are then counted. Each colony corresponds to a single viable cell, providing an estimate of the bacterial concentration in the sample.

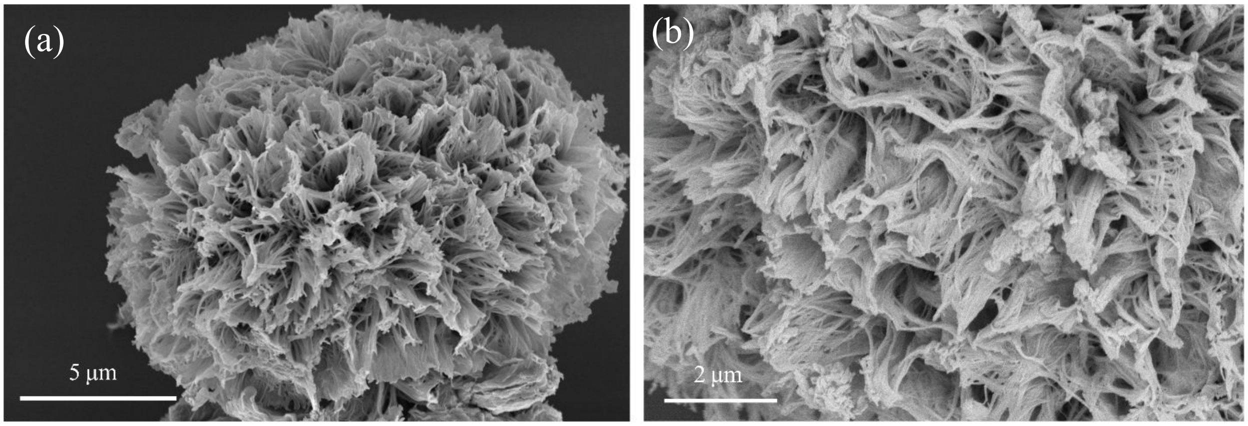

Figure S4. Typical SEM images of K-1DL PMPs. Morphology is similar to that of its Na-counterpart shown in Figure 1c.

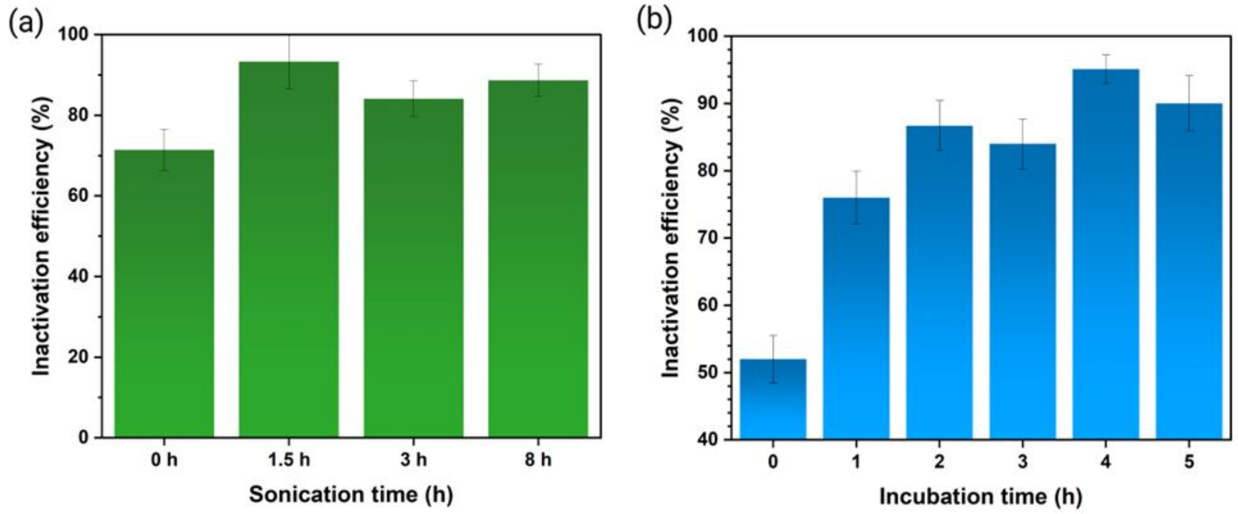

Figure S5. (a) Inactivation efficiency of TMA-1DL (1000 µg/mL) against *E. coli* (final concentration of 10^6 CFU/mL) after 3 h of incubation, sonicated for the indicated durations (0, 1.5, 3, and 8 h). (b) *E. coli* bacterial cells were recultivated after treatment with 1000 µg/mL of TMA-1DL for 0 to 5 h incubation time.

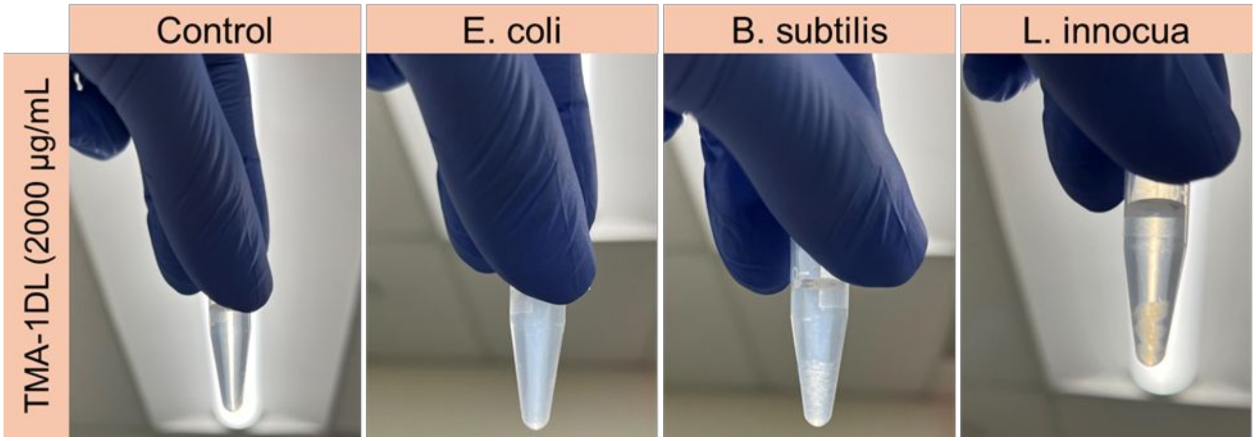

Figure S6. Optical images of *E. coli*, *B. subtilis*, and *L. innocua* bacterial cells (final concentration 10^6 CFU/mL) re-cultivated after 4 h of treatment with 2000 µg/mL TMA-1DL, showing bacterial agglomeration. Bacterial suspensions (final concentration 10^6 CFU/mL) in DI water without TMA-1DL served as a control.

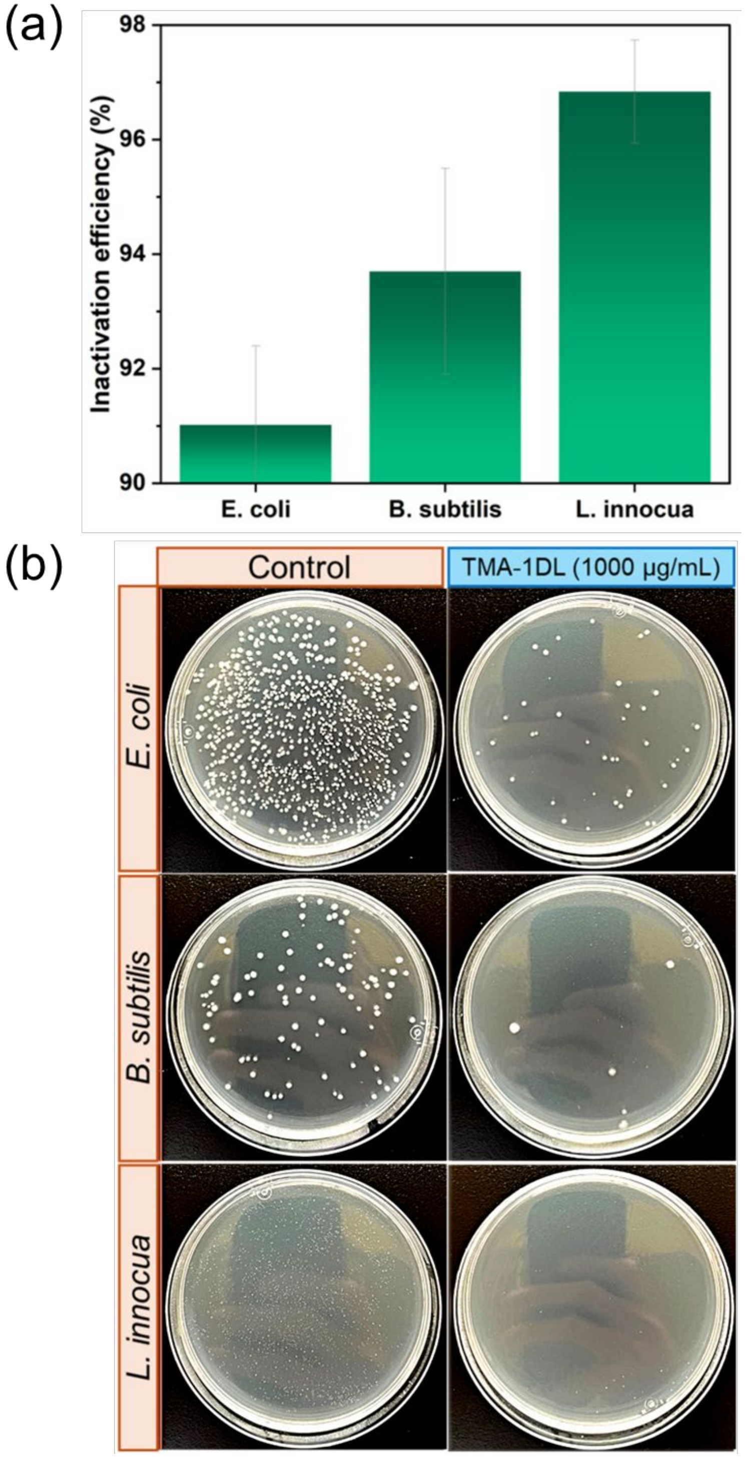

Figure S7. (a) Antibacterial activity of TMA-1DL (1000 µg/mL) against *E. coli*, *B. subtilis*, and *L. innocua* bacterial cells (final concentration 10^6 CFU/mL) for 4 h in DI water instead of PBS. (b) Photographs of corresponding agar plates. Bacterial suspensions (final concentration 10^6 CFU/mL) in DI water without TMA-1DL material served as controls.

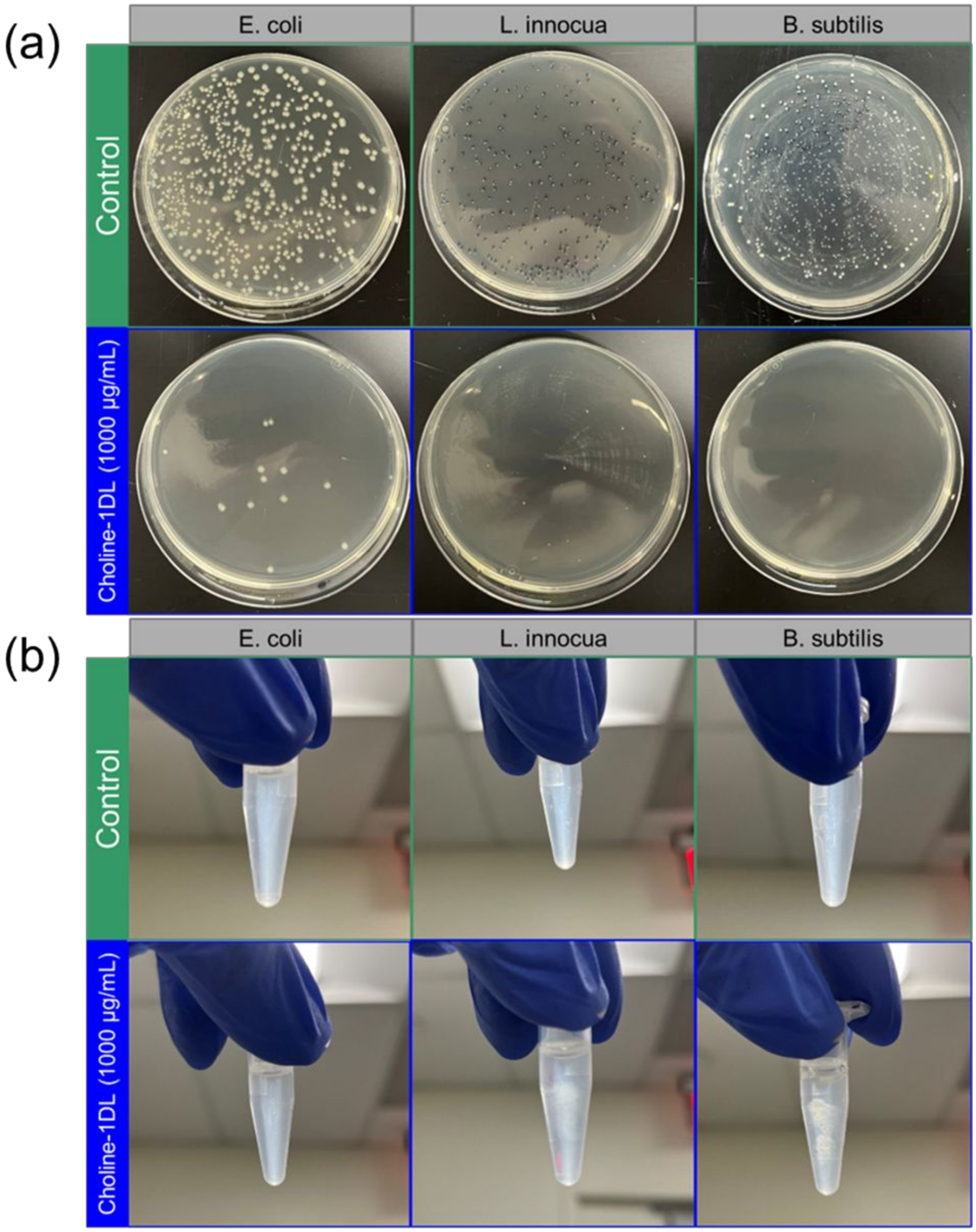

Figure S8. (a) Photographs of agar plates and (b) optical images of *E. coli*, *B. subtilis*, and *L. innocua* bacterial cells (final concentration 10^6 CFU/mL) recultivated after 4 h of treatment with 1000 µg/mL Cho-1DL. Bacterial suspensions (final concentration 10^6 CFU/mL) in DI water without Cho-1DL served as controls.

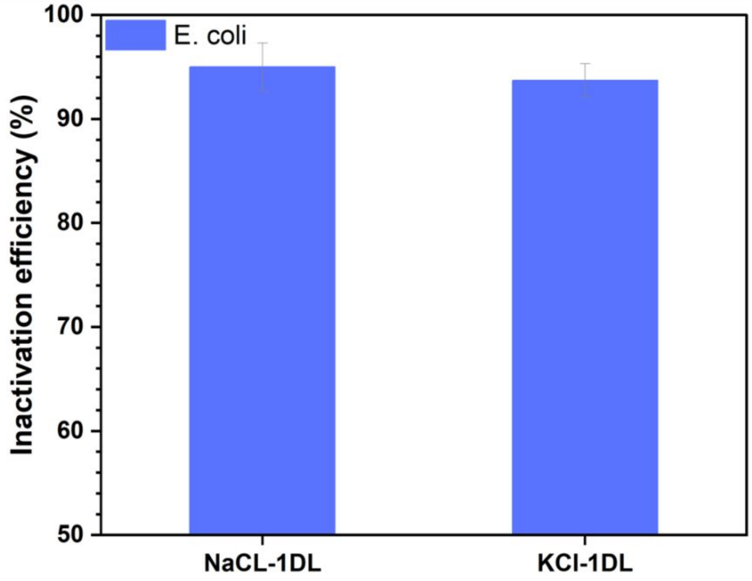

Figure S9. Antibacterial activities of NaCl-1DL and KCl-1DL PMPs. *E. coli* bacterial cells (final concentration 10^6 CFU/mL) were recultivated with 1000 μg/mL NaCl-1DL and KCl-1DL materials for 4 h of incubation.

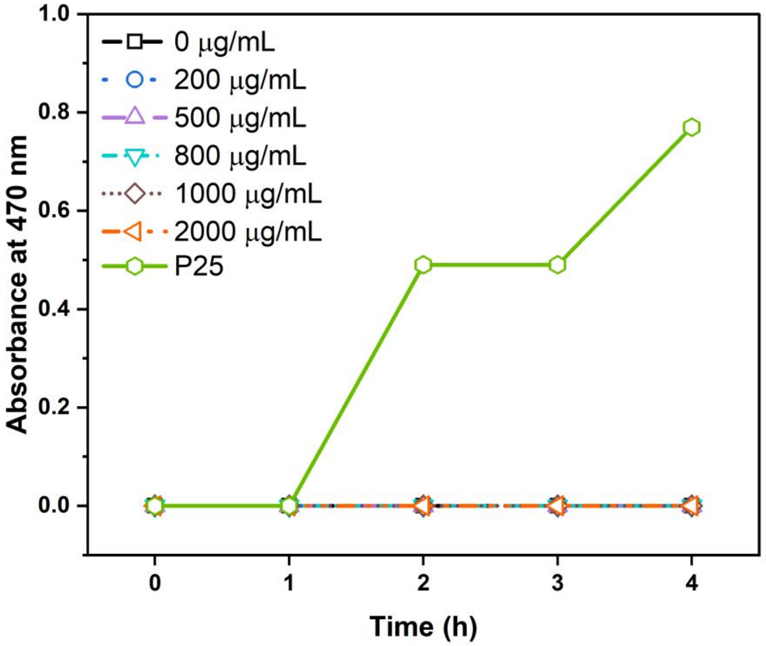

Figure S10. Formation of superoxide radicals ($O^{2\bullet-}$) evaluated by the XTT reduction assay in the dark. XTT (0.4 mM, pH 7.0) was incubated with 1DL NF colloidal suspensions at various concentrations for up to 4 h. Commercial P25 TiO_2 (1000 μg mL^{-1}) was used as a positive control.

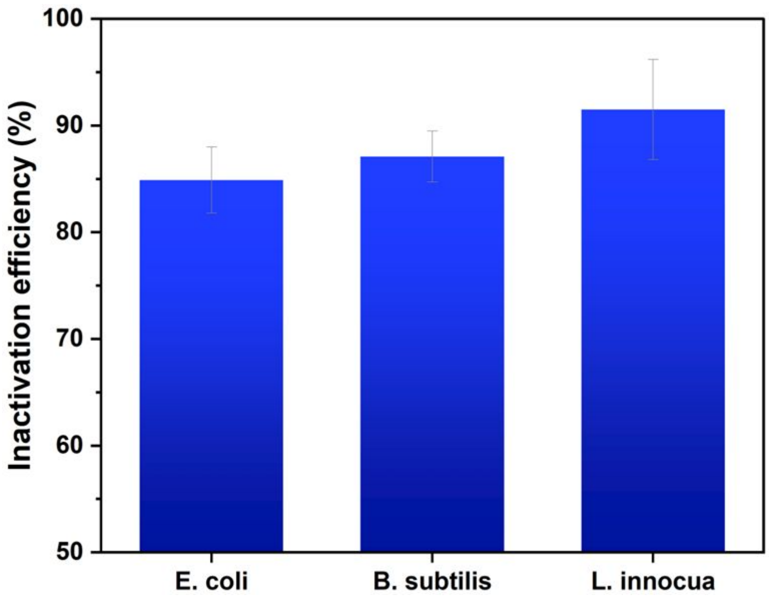

Figure S11. Percentage of dead/damaged bacterial cells for each strain after 4 h in the dark, as determined by flow cytometry.

Table S1. Comparison of antibacterial activities of titanate nanofilaments with those of TiO_2-based materials reported in the literature.

Material	Crystal form	Conc. (μg/mL)	Bacteria Conc. (CFU/mL)	Condition	Bacteria reduction (%)			Ref.
					E. coli	*B. subtilis*	*L. innocua*	
TiO_2	Anatase	1000	10^4	UV-light	98.5	-	-	2
TiO_2	Rutile	1000	10^4	UV-light	93.7	-	-	2
TiO_2	Anatase	500	10^6	Normal light	97	-	-	3
P25 TiO_2	Anatase	200	10^8	Normal light	75	-	-	4
	Anatase	100	$6 * 10^5$	Normal light	45.6	-	-	5
TiO_2	Anatase	100	$6 * 10^5$	Normal light	88.2	-	-	5
TiO_2	Anatase	200	10^8	UV-light	99	-	-	6
TiO_2	Anatase	1000	10^7	Dark	69.6	-	-	7
TiO_2	Anatase	1000	10^7	UV-light	98.2	-	-	7
TiO_2	Anatase	5000	10^7	UV-light	89.1	93	-	8
TiO_2	Anatase	150	10^6	Dark	-	63	-	9
TiO_2	Anatase/rutile	1000	10^8	UV-light	-	98	-	10
TiO_2	-	100	10^6	Normal light	83.7	93.6	-	11
TiO_2	Anatase	2000	10^8	Normal light	80	90	-	12
Titanate Nanofiber*	N/A	1000	10^7	UV-light	99	-	-	13
Titanate nanofilament	TMA-Lepidocrocite	1000	10^6	Normal light	96.2	98.9	99.8	This work
	Chol-Lepidocrocite			Normal light	96.8	99.7	99.2	
	TMA-Lepidocrocite			Dark	84.9	87.1	91.5	

*The incubation time is 24 hours.

References

(1) Ibrahim, M. A.; Walter, A. D.; Badr, H. O.; Schwenk, G. R.; Ibrahim, A. M. H.; Morris, V. R.; Boukhris, S.; Florea, M.; Constantin, D.; Barsoum, M. W. Expanding the processing space of quantum confined, one-dimensional titania-based lepidocrocite nanofilaments. *Matt.* **2025**, *8* (7). DOI: 10.1016/j.matt.2025.102260 (acccessed 2025/11/07).
(2) Liao, C.; Li, Y.; Tjong, S. C. Visible-Light Active Titanium Dioxide Nanomaterials with Bactericidal Properties. *Nanomater.* **2020**, *10* (1), 124.
(3) Lin, X.; Li, J.; Ma, S.; Liu, G.; Yang, K.; Tong, M.; Lin, D. Toxicity of TiO2 nanoparticles to Escherichia coli: effects of particle size, crystal phase and water chemistry. *PLoS One* **2014**, *9* (10), e110247.
(4) Zimbone, M.; Buccheri, M.; Cacciato, G.; Sanz, R.; Rappazzo, G.; Boninelli, S.; Reitano, R.; Romano, L.; Privitera, V.; Grimaldi, M. Photocatalytical and antibacterial activity of TiO2 nanoparticles obtained by laser ablation in water. *Appl. Catal. B: Environ.* **2015**, *165*, 487-494.
(5) Tang, Y.; Sun, H.; Shang, Y.; Zeng, S.; Qin, Z.; Yin, S.; Li, J.; Liang, S.; Lu, G.; Liu, Z. Spiky nanohybrids of titanium dioxide/gold nanoparticles for enhanced photocatalytic degradation and anti-bacterial property. *J. Colloid. Interface Sci.* **2019**, *535*, 516-523.
(6) Zhukova, L. V. Evidence for Compression of Escherichia coli K12 Cells under the Effect of TiO2 Nanoparticles. *ACS Appl. Mater. Interfaces.* **2015**, *7* (49), 27197-27205. DOI: 10.1021/acsami.5b08042.
(7) Leung, Y. H.; Xu, X.; Ma, A. P. Y.; Liu, F.; Ng, A. M. C.; Shen, Z.; Gethings, L. A.; Guo, M. Y.; Djurišić, A. B.; Lee, P. K. H.; et al. Toxicity of ZnO and TiO2 to Escherichia coli cells. *Sci. Rep.* **2016**, *6* (1), 35243.
(8) Park, S.; Lee, S.; Kim, B.; Lee, S.; Lee, J.; Sim, S.; Gu, M.; Yi, J.; Lee, J. Toxic effects of titanium dioxide nanoparticles on microbial activity and metabolic flux. *Biotechnol. Bioprocess Eng.* **2012**, *17*, 276-282.
(9) Akhtar, S.; Shahzad, K.; Mushtaq, S.; Ali, I.; Rafe, M. H.; Fazal-ul-Karim, S. M. Antibacterial and antiviral potential of colloidal Titanium dioxide (TiO2) nanoparticles suitable for biological applications. *Mater. Res. Express* **2019**, *6* (10), 105409.
(10) Li, M.; Noriega-Trevino, M. E.; Nino-Martinez, N.; Marambio-Jones, C.; Wang, J.; Damoiseaux, R.; Ruiz, F.; Hoek, E. M. Synergistic bactericidal activity of Ag-TiO2 nanoparticles in both light and dark conditions. *Environ. Sci. Technol.* **2011**, *45* (20), 8989-8995.
(11) Anbumani, D.; Dhandapani, K. v.; Manoharan, J.; Babujanarthanam, R.; Bashir, A. K. H.; Muthusamy, K.; Alfarhan, A.; Kanimozhi, K. Green synthesis and antimicrobial efficacy of titanium dioxide nanoparticles using Luffa acutangula leaf extract. *J. King Saud Univ. Sci.* **2022**, *34* (3), 101896.
(12) Sondezi, N.; Njengele-Tetyana, Z.; Matabola, K. P.; Makhetha, T. A. Sol–gel-derived TiO2 and TiO2/Cu nanoparticles: synthesis, characterization, and antibacterial efficacy. *ACS Omega* **2024**, *9* (14), 15959-15970.
(13) Yada, M.; Inoue, Y.; Noda, I.; Morita, T.; Torikai, T.; Watari, T.; Hotokebuchi, T. Antibacterial Properties of Titanate Nanofiber Thin Films Formed on a Titanium Plate. *J. Nanomater.* **2013**, *2013* (1), 476585. DOI: https://doi.org/10.1155/2013/476585.
